# Castor Oil for Induction of Labor: A Safe and Effective Method: A Large Retrospective Cohort Study in a University Hospital Setting

**DOI:** 10.3390/healthcare14040496

**Published:** 2026-02-14

**Authors:** Peter Jakubowski, Dorothée Hoffmann, Birgitt Schönfisch, Harald Abele, Jürgen Andress, Kristina Bettecken

**Affiliations:** 1 Department for Women’s Health, University Hospital of Tübingen, Calwerstrasse 7, 72076 Tübingen, Germany; 2 Research Institute for Women’s Health, University of Tübingen, 72076 Tübingen, Germany; 3 Section of Midwifery Science, Institute for Health Sciences, University Hospital of Tübingen, Calwerstrasse 7, 72076 Tübingen, Germany

**Keywords:** castor oil, induction, labor, prostaglandins, oxytocin, uterine rupture

## Abstract

**Background/Objectives:** Castor oil has long been used as a traditional method for inducing labor, but evidence regarding its safety and effectiveness remains limited. This study aimed to assess the efficacy and safety of castor oil for labor induction in a large cohort within a high-risk university hospital setting. **Methods:** A retrospective analysis was conducted on women who underwent labor induction between January 2017 and June 2018 at the Department for Women’s Health, University Hospital Tübingen. Outcomes of women induced primarily with castor oil-based induction cocktail and their offspring were compared with those induced using standard methods (prostaglandins, oxytocin, ripening balloon). Primary outcomes included induction-to-delivery time, need for additional induction methods, mode of delivery, and maternal/fetal complications. **Results:** A total of 1015 women were included; 824 (82.1%) received castor oil, and 191 (18.8%) underwent standard induction. The mean induction-to-delivery interval was 26.9 h in the castor oil group versus 19.0 h in the standard group. Additional induction was required in 50.5% of the castor oil group compared to 37.2% of the standard group. No significant differences were observed between groups regarding delivery mode or maternal/fetal complications. **Conclusions:** Castor oil appears to be a safe but slightly less effective option for inducing labor close to the calculated due date, even in a high-risk population. Large-scale prospective randomized trials are warranted to further evaluate its role in clinical practice.

## 1. Introduction

Induction of labor is among the most common obstetric interventions, with approximately 20% of pregnant women undergoing this procedure—and up to 25% in high-risk settings [1,2]. It is indicated when a planned vaginal birth appears more beneficial than expectant management or cesarean section. Determining the appropriate method for induction is challenging for obstetricians, as it requires balancing medical considerations with the individual preferences, wishes, and concerns of the expectant mother.

In 2020 and again in 2025, public debate in Germany surrounding the use of misoprostol (Cytotec^®^ and Angusta^®^) for labor induction resurfaced, leading to widespread skepticism about this method [3,4]. Many women expressed a desire for a non-pharmacological approach to induction.

Castor oil has been used for centuries to induce labor, although clinical evidence remains limited. Early references to its use date back more than 400 years, with anecdotal reports even tracing its origins to ancient Egypt [5,6]. The laxative effect of castor oil stimulates intestinal activity. After emulsification by pancreatic lipases and bile, it is converted into ricinoleic acid, which exerts a laxative effect. Furthermore, ricinoleic acid binds to prostaglandin receptors in smooth muscle tissue, promoting the production of prostaglandin E2 [7]. Common side effects include diarrhea and nausea [5].

Current clinical guidelines do not recommend the use of castor oil for labor induction due to insufficient evidence regarding its safety and efficacy, or only under study conditions [5,8,9]. A recurring issue in the literature is the considerable variation in the administered dose of castor oil, which ranges from 20 to 60 mL depending on the study [9]. However, a recent retrospective German study suggested that castor oil may be both safe and effective for multiparous women [10].

This retrospective study aimed to demonstrate the efficacy and safety of castor oil as part of an “induction cocktail” for labor induction in a tertiary-care obstetric population (university hospital). Women with a history of cesarean section were also included.

## 2. Material and Methods

### 2.1. Study Design

We conducted a retrospective data analysis of all women with viable singleton pregnancies who underwent labor induction between January 2017 and June 2018 at the Department for Women’s Health, University Hospital Tübingen, Germany (Perinatal Center Level 1; tertiary care center).

Women with multiple pregnancies or intrauterine fetal demise were excluded from the analysis.

Two groups were defined: (1) women receiving castor oil as a first-line method for labor induction (the cocktail group) usually offered from 38 + 0 weeks of gestation onwards, and (2) a reference group of women induced using standard methods (i.e., prostaglandins, oxytocin, or a cervical ripening balloon (CRB).

Primary outcome variables included the number of induction attempts required, time from induction to delivery, duration of hospital stay, mode of delivery (spontaneous vaginal, vacuum/forceps-assisted, or cesarean section), complications (postpartum hemorrhage, curettage, uterine rupture), 5-min Apgar score, and umbilical arterial cord pH.

Potential influencing variables were maternal age, body mass index (BMI), parity, gestational age, rupture of membranes, indication for induction, Bishop score, number of contractions per 30 min prior to induction, and use of castor oil for induction.

### 2.2. Data Collection and Statistical Analysis

Patient data were extracted from clinical records (SAP ISH-med module) and entered into a REDCap database. All data were pseudonymized prior to analysis. Data processing and statistical analyses were performed using Microsoft Excel and the software R (version 4.2.2).

Descriptive univariate analyses were conducted. Statistical significance was assessed using the *t*-test respectively the Wilcoxon-Mann-Whitney rank test for quantitative variables, and Fisher’s exact test for qualitative variables. A *p*-value < 0.05 was considered statistically significant.

### 2.3. Induction Protocol

Generally, women scheduled for labor induction after 38 + 0 weeks of gestation were offered a castor oil cocktail as a first-line intervention. If castor oil was either declined or contraindicated (e.g., in women with chronic inflammatory bowel disease), induction was carried out using standard methods—prostaglandins (50 µg vaginal misoprostol, 1–2 mg dinoprostone vaginal gel, or rarely a misoprostol vaginal insert), oxytocin, or, in rare cases, a CRB. One induction attempt was defined as one single administration of an induction agent.

If labor did not commence following castor oil administration, a subsequent induction was initiated with standard induction methods the next day.

The protocols for standard induction methods were as follows:

- 50 µg misoprostol was applied vaginally up to four times daily at 4–6-h intervals.
- 1 mg or 2 mg dinoprostone vaginal gel was administered approximately twice daily.
- The misoprostol vaginal insert (misodel) was applied for 12 h, followed by at least a 12-h pause before further induction. This method was only used in five cases.
- The CRB was inserted for 12 h.

The induction cocktail contained the following standardized ingredients: 20 mL castor oil, 1 tablespoon of almond butter, 150 mL apricot juice, a dash of cinnamon, and 200 mL of non-alcoholic sparkling wine or sparkling water. Women were instructed to consume the cocktail within 20–30 min (Table 1).

This study was approved by the ethical committee of the University of Tübingen (AZ: 651/2019BO2).

## 3. Results

### 3.1. Patient Characteristics

A total of 1015 women were included in our study who gave birth between January 2017 and June 2018. Of these, 824 women (81.2%) received an induction cocktail as the first-line method. Patient characteristics are summarized in Table 2.

Women who received the induction cocktail did not differ significantly in age or BMI before pregnancy compared with the reference group. Women in the cocktail group had significantly less favorable Bishop scores (*p* < 0.001) and were at a significantly higher gestational age at the time of induction (*p* < 0.001). They also exhibited fewer uterine contractions on the cardiotocography (CTG) prior to induction (*p* = 0.011).

However, a significantly higher proportion of nulliparous women received an induction cocktail compared with multiparous women (84.8% vs. 76.3%; *p* < 0.001). 105 women underwent a after previous cesarean (TOLAC). All other previous birth modes where spontaneous or vaginal-operative (Table 3).

### 3.2. Indications

The most common indications for induction were (in descending order) premature rupture of membranes, oligohydramnios, post-term pregnancy, diabetes, gestational hypertension/preeclampsia, abnormal fetal heart rate patterns assessed by cardiotocography (CTG), small for gestational age/fetal growth restriction (SGA/FGR), decreased fetal movements, and other causes (Table 4).

### 3.3. Number of Inductions Required

Among the 824 women in the cocktail group, 408 (49.5%) did not require any further induction. In the reference group, 120 women (62.8%) went into labor after a single induction. The median number of inductions was 2 in the cocktail group and 1 in the reference group. Women undergoing TOLAC went into labor after the cocktail only in 39 (45.3%) and in 7 (36.8%) cases (Table 5).

Women receiving the induction cocktail as the first-line method required significantly more cumulative inductions than those treated with conventional methods (*p* < 0.001). Nearly half of the women in the cocktail group entered labor without the need for any additional induction measures.

### 3.4. Time to Delivery, Mode of Birth, and Hospital Stay

The mean time from the first induction to delivery was significantly longer in the cocktail group compared with the reference group (26.9 vs. 19.0 h, *p* < 0.001), also in the patients receiving only one induction agent (12.8 vs. 9.8 h, *p* < 0.001) (Table 6).

Although proportions of vaginal birth mode are quite (12.9% vs. 7.9%) there were no significant differences in the distribution of birth modes (spontaneous vaginal, operative vaginal, unplanned cesarean section, or emergency cesarean section) between the groups (Table 7, *p* = 0.229).

A total of 105 women underwent TOLAC (Table 8). Within this subgroup, no significant differences in delivery modes were observed (*p* = 0.817). However, due to the small numbers of the subgroup, the statistical power in this analysis was limited.

The total duration of hospital stay did not differ significantly between the two groups (Table 9, *p* = 0.778).

### 3.5. Fetal Outcomes and Maternal Complications

The umbilical arterial pH values did not differ significantly between the groups (Table 10, *p* = 0.239).

Similarly, the proportion of patients with complications, including postpartum hemorrhage, curettage, and uterine rupture, did not differ significantly between groups (Table 10, *p* = 1.000).

### 3.6. Uterine Ruptures

In the entire study population, two cases of uterine rupture were observed in women giving birth after a previous cesarean section: one complete rupture and one rupture covered by the urinary bladder (dehiscence).

In the first case, a complete uterine rupture occurred following induction with the castor oil cocktail only. An emergency cesarean section was performed at full cervical dilation due to pathological CTG findings and maternal suprapubic pain. The neonate had Apgar scores of 2/7/9 and an initial umbilical artery pH of 6.98. The fetal head was impacted, and intraoperative findings confirmed a complete uterine rupture. The newborn was admitted to the neonatal intensive care unit (NICU) for two days, with no indication for hypothermia therapy. Follow-up examinations up to three years of age revealed no signs of developmental impairment.

In the second case, a contained uterine rupture (dehiscence) occurred after induction with the castor oil cocktail only. An unscheduled cesarean section was performed for arrest of labor at full cervical dilation. CTG findings remained normal throughout labor. Intraoperatively, a uterine rupture covered by the bladder was identified. The neonate had Apgar scores of 10/10/10 and an umbilical artery pH of 7.19.

## 4. Discussion

### 4.1. Overall Findings

Our data suggest that the castor oil cocktail seems to be a safe and effective method for induction of labor. In our study population it led to the onset of labor in nearly 50% of women, even in a high-risk, tertiary-care setting. This study includes a large proportion of primiparous women (57% of cases), women with unfavorable Bishop scores (<6 in 97%), and with previous cesarean sections (10.3%). To our knowledge, this retrospective cohort represents the largest study of its kind published to date. Previous randomized controlled trials investigating castor oil for labor induction have included relatively small cohorts, typically fewer than 200 women, and focused mainly on post-term pregnancies [11,12]. In a meta-analysis by Moradi et al., only two of eight included studies were placebo-controlled [11].

A recent review by Sanchez-Ramos et al. argued that castor oil is no longer used for labor induction in hospital settings in high-income countries following the introduction of pharmacological induction agents [13]. We disagree with this assessment, as our findings—and those reported by Ziegler et al. [10]—demonstrate that castor oil remains a clinically applicable and effective option. Ziegler et al. offered castor oil for labor induction to multiparous women in a university hospital setting and found it to be effective, although their study excluded nulliparous women [10].

### 4.2. Effectiveness of the Castor Oil Cocktail

In our study, the onset of labor occurred in 49.5% of women after administration of the castor oil cocktail alone. The mean time interval from induction to birth was 26.9 (±21.7) hours. In a randomized controlled trial comparing oral and vaginal misoprostol with vaginal dinoprostone gel, the mean induction-to-delivery interval ranged from 20.1 (±10.2) hours for dinoprostone gel to 22.6 (±17.2) hours for oral misoprostol and 25.5 (±54.2) hours for vaginal misoprostol [14]. In our reference group, which primarily included women receiving vaginal dinoprostone or misoprostol, the mean induction-to-delivery time was 19.0 (±20.5) hours. Although this interval was significantly shorter than in the cocktail group, the fact that nearly half of the women in the latter achieved labor onset without additional induction measures and within 27 h supports the clinical effectiveness of the method, particularly given the high-risk nature of the population and less favorable Bishop scores.

Our results on the effectiveness are further supported by a meta-analysis which showed a significantly higher rate of onset of labor within 24 h after administration of castor oil, compared to the control groups [12].

Parity and cervical status at baseline likely contributed substantially to these differences in the time from induction to birth. This interpretation is consistent with findings from a recent German study, which reported that 74% of multiparous women entered labor after a single administration of a castor oil cocktail [10]. Taken together, these data support the effectiveness of castor oil as an induction agent in both multiparous and, to a lesser extent, nulliparous women.

Interpretation of these results must account for baseline differences between groups. These are due to the clinic’s protocol of offering all women the inductions cocktail as primary induction method. Women in the cocktail group had a higher gestational age, less favorable Bishop scores, fewer uterine contractions prior to induction, and a higher proportion of nulliparous women. These factors are known to be associated with a longer induction process and lower induction success rates. These characteristics likely contributed to the longer induction-to-delivery interval observed. Against this background, the achievement of labor onset in nearly half of the women following the castor oil cocktail alone supports its clinical effectiveness even under less favorable conditions.

### 4.3. Safety of the Castor Oil Cocktail

No significant differences were observed between the groups in maternal or neonatal complications. The overall rate of complications was low, including postpartum hemorrhage (*n* = 15), curettage (*n* = 36), and uterine rupture/dehiscence (*n* = 2). The incidence of severe fetal acidosis (pH < 7.0) was also consistently low in both groups (0.6% vs. 0.5%). These findings align with previous studies demonstrating no significant differences in cesarean section rates, instrumental delivery, meconium-stained amniotic fluid, or low Apgar scores between castor oil and placebo or no-treatment groups [5]. Furthermore, both observational and randomized studies have shown no increased risk of fetal distress, uterine rupture, abnormal maternal blood pressure, postpartum hemorrhage, or neonatal resuscitation associated with castor oil use [15,16,17].

### 4.4. Uterine Rupture and Dehiscence

Uterine rupture is a potentially life-threatening complication in women with prior cesarean delivery, associated with increased maternal and neonatal morbidity and mortality. It is defined as a complete disruption of the myometrium and visceral peritoneum, whereas uterine dehiscence describes myometrial disruption with intact serosa [18]. The estimated rate of uterine rupture among women undergoing TOLAC is approximately 0.47% [19], and induction of labor has been associated with an almost threefold increased risk (OR 2.86; 95% CI 1.75–4.67) [18]. After pharmacological induction, the risk of rupture is estimated at 1–1.5%, or roughly one in every 75–100 TOLAC cases [18,19].

Only two prior studies have investigated castor oil use in TOLAC settings. Fruscalzo et al. permitted only amniotomy or castor oil for induction in women with prior cesarean delivery, reporting one case of symptomatic rupture among 170 cases, without specifying which induction method was used [20]. Bayoumi et al. conducted a randomized, double-blind trial in 70 TOLAC patients comparing 60 mL castor oil with sunflower oil as placebo. Labor onset occurred in 45.7% of the intervention group versus 8.5% in the placebo group, and 65.7% achieved successful TOLAC within one week. No cases of uterine rupture were reported, likely due to the limited sample size [16].

In our study, one complete uterine rupture and one contained rupture (dehiscence) occurred following induction with the castor oil cocktail alone. These rates are comparable to those reported in the literature following pharmacological induction [18,19]. Although our sample size is insufficient to draw definitive conclusions, the very low incidence of uterine rupture supports the safety of castor oil induction even in a high-risk population. Nonetheless, continuous monitoring of labor progression and fetal heart rate is essential, and induction should only be performed in facilities equipped for immediate emergency cesarean delivery [9]. We do not recommend the use of castor oil for labor induction outside of a hospital setting.

### 4.5. Strengths and Limitations

This study has several limitations that should be considered when interpreting the results. First, due to its retrospective, single-center design, the findings are descriptive and exploratory in nature rather than comparative, and no causal inferences can be drawn. This design may also limit the generalizability of the results to other clinical settings, including lower-risk or community hospitals. Second, the control group was not matched, which may have introduced confounding and limits the validity of direct comparisons between induction methods. A further limitation is the marked imbalance in sample size between the induction cocktail and reference groups. This reflects routine clinical practice at our institution, where the castor oil cocktail was predominantly used as a first-line induction method during the study period. Accordingly, the analyses were descriptive and exploratory rather than comparative. Statistical methods appropriate for unbalanced groups were applied, and clinically robust outcomes were examined. While the unequal group sizes may limit statistical precision, the consistent findings across key maternal and neonatal outcomes support the internal validity of the results.

Third, although the overall sample size was substantial, the subgroup of women undergoing TOLAC was relatively small (*n* = 105; 86 in the induction cocktail group and 19 in the reference group). Given the expected uterine rupture rate of 1–1.5% in TOLAC, the study was underpowered to detect statistically significant differences in this rare but clinically important outcome.

Fourth, the castor oil induction protocol used in this study employed a lower dose (20 mL) compared with other studies, which commonly used up to 60 mL [11,12]. Patient-reported outcomes, such as satisfaction and side effects of castor oil administration, were also not systematically assessed, which could represent an additional limitation. Finally, the induction cocktail contained multiple components (almond butter, apricot juice, cinnamon, and sparkling wine or water) in addition to castor oil, preventing isolation of the specific effects of castor oil. Variability in dosing and administration of standard induction methods may also have introduced additional confounding.

Despite these limitations, this study has several important strengths. It represents the largest retrospective evaluation to date of castor oil–based induction of labor in a high-risk, tertiary-care setting and includes both nulliparous and multiparous women, as well as women undergoing TOLAC. The study provides comprehensive, real-world clinical data with detailed maternal and neonatal outcomes, thereby contributing valuable evidence on the safety and effectiveness of castor oil induction in contemporary obstetric practice. Nevertheless, the findings should be considered hypothesis-generating and warrant confirmation in prospective, balanced studies.

## 5. Conclusions

Castor oil for induction of labor seems to be a safe and promising method with a relatively high success rate, even in a high-risk population. The rate of complications seems to be comparable to established methods of induction. Considering the potential risks and complications associated with the use of castor oil for labor induction, its administration should be confined to a clinical setting with the necessary resources and expertise to ensure appropriate management, including adequate maternal and fetal monitoring.

## Figures and Tables

**Table 1:** Induction Cocktail Recipe.

Induction Cocktail Recipe	
Castor oil	20 mL
Almond butter	1 Tablespoon
Apricot juice	150 mL
Cinnamon	1 dash
Sparkling wine (non-alcoholic) or sparkling water	200 mL
Consume within 20–30 min	

**Table 2:** Patient Characteristics.

	All (*n* = 1015)	Cocktail (*n* = 824)	Reference (*n* = 191)	*p* Value
Maternal age (years, mean ± SD, range)	31.9 ± 5.0 16.4–46.8	32.0 ± 5.1 16.6–46.8	31.6 ± 4.9 16.4–41.6	0.303 ^a^
BMI before pregnancy ⱡ (kg/m^2^, mean ± SD, range)	25.6 ± 5.9 16.8–63.5	25.5 ± 5.7 16.8–63.5	25.8 ± 6.4 16.8–51.4	0.799 ^b^
Gestational age at induction (days ± SD, range)	277 ± 10 238–294	280 ± 8 259–294	268 ± 13 238–293	<0.001 ^b^
Bishop Score before induction (number of patients)				
0	744 (73.3%)	650 (78.9%)	94 (49.2%)	<0.001 ^c^
1–5	244 (24.0%)	169 (20.5%)	75 (39.3%)	
≥6	24 (2.4%)	2 (0.3%)	22 (11.5%)	
unknown	3 (0.3%)	3 (0.4%)	0 (0.0%)	
Contractions/30 Minutes before first induction				
0	831 (81.9%)	687 (83.4%)	144 (75.4%)	0.011 ^c^
1–3	172 (16.9%)	130 (15.8%)	42 (22.0%)	
4–6	12 (1.2%)	7 (0.8%)	5 (2.6%)	

^a^ = *t*-Test, ^b^ = Wilcoxon-Mann-Whitney rank test, ^c^ = Fisher’s exact test, ⱡ one value unknown.

**Table 3:** History of Birth Mode.

Number of Patients	All (*n* = 1015)	Cocktail (*n* = 824)	Reference (*n* = 191)	*p* Value
Previous deliveries				
0	580 (57.1%)	492 (59.7%)	88 (46.1%)	<0.001 ^c^
≥1	435 (42.9%)	332 (40.3%)	103 (53.9%)	
Previous cesarean section	105 (10.3%)	86 (10.4%)	19 (9.9%)	

^c^ = Fisher’s exact test.

**Table 4:** Indication for Induction.

Indication for Induction (Multiple Indications Possible)	All (*n* = 1015)	Cocktail (*n* = 824)	Reference (*n* = 191)
Premature Rupture of membranes	265 (26.1%)	189 (22.9%)	76 (39.8%)
Oligohydramnion	243 (23.9%)	220 (26.7%)	23 (12.0%)
Post-term pregnancy	212 (20.9%)	195 (23.7%)	17 (8.9%)
Diabetes mellitus	135 (13.3%)	116 (14.1%)	19 (9.9%)
Gestational hypertension/ preeclampsia	102 (10.0%)	71 (8.6%)	31 (16.2%)
Abnormal fetal heart rate patterns	99 (9.8%)	82 (10.0%)	17 (8.9%)
SGA/FGR	90 (8.9%)	80 (9.7%)	10 (5.2%)
Decreased fetal movements	75 (7.4%)	52 (6.3%)	23 (12.0%)
Fetal malformation	44 (4.3%)	37 (4.5%)	7 (3.7%)
Pathological Doppler ultrasound	36 (3.5%)	27 (3.3%)	9 (4.7%)
Maternal medical conditions	32 (3.2%)	21 (2.5%)	11 (5.8%)
Others	76 (7.5%)	38 (4.6%)	38 (19.9%)

**Table 5:** Cumulative Number of Inductions Required.

Number of Patients	Number of Inductions Required									*n*
	1	2	3	4	5	6	7	8	9	
All	528	185	222	26	32	8	9	3	2	1015
Cocktail	408	147	204	23	24	6	9	1	2	824
Reference	120	38	18	3	8	2	0	2	0	191
TOLAC	46	17	29	6	2	1	2	1	1	105
Cocktail TOLAC	39	10	26	6	1	1	2	0	1	86
Reference TOLAC	7	7	3	0	1	0	0	1	0	19

**Table 6:** Time Between Induction and Birth.

	Time Between Induction and Birth [Hours]						
	Mean	SD	Median	Min	Max	*n*	*p* Value
All	25.4	21.7	21.2	0.9	204.7	1015	
Cocktail	26.9	21.7	24.1	0.9	204.7	824	<0.001
Reference	19.0	20.5	11.6	1.2	127.7	191	
Cocktail, one induction only	12.8	7.8	10.9	0.9	60	408	<0.001
Reference, one induction only	9.8	6.8	8.2	1.2	35.5	120	

**Table 7:** Birth Mode.

Number of Patients	Spontaneous	Cesarean Section	Emergency Cesarean Section	Vaginal Operative	*n*	*p* Value
All	653 (64.3%)	232 (22.9%)	9 (0.9%)	121 (11.9%)	1015	
Cocktail	522 (63.3%)	188 (22.8%)	8 (1.0%)	106 (12.9%)	824	0.229
Reference	131 (68.6%)	44 (23.0%)	1 (0.5%)	15 (7.9%)	191	

**Table 8:** Trial of Labor After Previous Cesarean.

Number of Patients	Spontaneous	Cesarean Section	Emergency Cesarean Section	Vaginal Operative	*n*	*p* Value
All	45 (42.9%)	46 (43.8%)	2 (1.9%)	12 (11.4%)	105	
Cocktail	35 (40.7%)	39 (45.3%)	2 (2.3%)	10 (11.6%)	86	0.817
Reference	10 (52.6%)	7 (36.8%)	0 (0.0%)	2 (10.5%)	19	

**Table 9:** Duration of Hospital Stay in Days.

Hospital Stay in Days	Mean	SD	Median	Min	Max	*n*	*p* Value
All	4.4	1.8	4.1	0.4	16.5	1015	
Cocktail	4.4	1.5	4.1	0.8	14.8	824	0.778
Reference	4.7	2.6	4.2	0.4	16.5	191	

**Table 10:** Fetal Outcome and Maternal Complications.

	All (*n* = 1015)	Cocktail (*n* = 824)	Reference (*n* = 191)	*p* Value
5-min APGAR (mean ± SD)	9.3 ± 0.9	9.3 ± 0.8	9.2 ± 1.0	0.167
Umbilical artery pH (mean ± SD)	7.23 ± 0.08	7.23 ± 0.08	7.24 ± 0.07	0.064
Umbilical artery pH (number (percentage))				
>7.20	649 (63.9%)	515 (62.5%)	134 (70.2%)	0.239
≤7.20 and >7.10	300 (29.6%)	254 (30.8%)	46 (24.1%)	
≤7.10 and >7.00	60 (5.9%)	50 (6.1%)	10 (5.2%)	
≤7.00	6 (0.6%)	5 (0.6%)	1 (0.5%)	
Any maternal complication (number of patients (percentage))	43 (4.2%)	35 (4.2%)	8 (4.2%)	1.000
Maternal complications (number (percentage)) (multiple complications possible)				
Postpartum hemorrhage	15 (1.5%)	12 (1.5%)	3 (1.6%)	n.a.
Curettage	36 (3.5%)	29 (3.5%)	7 (3.7%)	
Uterus rupture	2 (0.2%)	2 (0.2%)	0 (0.0%)	

n.a. = not applicable due to small sample size.

## Data Availability

The raw data supporting the conclusions of this article will be made available by the authors on request due to data protection regulations.

## Abbreviations

The following abbreviations are used in this manuscript:

AZ	Aktenzeichen (Ethical Approval Reference Number)
BMI	Body Mass Index
CI	Confidence Interval
CRB	Cervical Ripening Balloon
CTG	Cardiotocography
EGA	Estimated Gestational Age
FGR	Fetal Growth Restriction
NICU	Neonatal Intensive Care Unit
OR	Odds Ratio
PGE2	Prostaglandin E2
pH	Potential of Hydrogen
REDCap	Research Electronic Data Capture
SAP ISH-med	SAP Integrated Solution for Healthcare—Medical Module
SD	Standard Deviation
SGA	Small for Gestational Age
TOLAC	Trial of Labor After Cesarean
